# Stress-Induced Hyperglycemia in Diabetes: A Cross-Sectional Analysis to Explore the Definition Based on the Trauma Registry Data

**DOI:** 10.3390/ijerph14121527

**Published:** 2017-12-07

**Authors:** Cheng-Shyuan Rau, Shao-Chun Wu, Yi-Chun Chen, Peng-Chen Chien, Hsiao-Yun Hsieh, Pao-Jen Kuo, Ching-Hua Hsieh

**Affiliations:** 1 Department of Neurosurgery, Kaohsiung Chang Gung Memorial Hospital and Chang Gung University College of Medicine, Kaohsiung 833, Taiwan; ersh2127@cloud.cgmh.org.tw; 2 Department of Anesthesiology, Kaohsiung Chang Gung Memorial Hospital and Chang Gung University College of Medicine, Kaohsiung 833, Taiwan; shaochunwu@gmail.com; 3 Department of Plastic Surgery, Kaohsiung Chang Gung Memorial Hospital and Chang Gung University College of Medicine, Kaohsiung 833, Taiwan; libe320@yahoo.com.tw (Y.-C.C.); VENU_CHIEN@hotmail.com (P.-C.C.); sylvia19870714@hotmail.com (H.-Y.H.); bow110470@gmail.com (P.-J.K.)

**Keywords:** stress-induced hyperglycemia, diabetes mellitus, glycemic gap, hemoglobin A1c, mortality, stress hyperglycemia ratio

## Abstract

*Background:* The diagnosis of diabetic hyperglycemia (DH) does not preclude a diabetes patient from having a stress-induced hyperglycemic response. This study aimed to define the optimal level of elevated glucose concentration for determining the occurrence of stress-induced hyperglycemia (SIH) in patients with diabetes. *Methods:* This retrospective study reviewed the data of all hospitalized trauma patients, in a Level I trauma center, from 1 January 2009 to 31 December 2016. Only adult patients aged ≥20 years, with available data on serum glucose and glycated hemoglobin A1c (HbA1c) levels upon admission, were included in the study. Long-term average glucose levels, as A1c-derived average glucose (ADAG), using the equation, ADAG = ((28.7 × HbA1c) − 46.7), were calculated. Patients with high glucose levels were divided into three SIH groups with diabetes mellitus (DM), based on the following definitions: (1) same glycemic gap from ADAG; (2) same percentage of elevated glucose of ADAG, from which percentage could also be reflected by the stress hyperglycemia ratio (SHR), calculated as the admission glucose level divided by ADAG; or (3) same percentage of elevated glucose as patients with a defined SIH level, in trauma patients with and without diabetes. Patients with incomplete registered data were excluded. The primary hypothesis of this study was that SIH in patients with diabetes would present worse mortality outcomes than in those without. Detailed data of SIH in patients with diabetes were retrieved from the Trauma Registry System. *Results:* Among the 546 patients with DH, 332 (32.0%), 188 (18.1%), and 106 (10.2%) were assigned as diabetes patients with SIH, based on defined glucose levels, set at 250 mg/dL, 300 mg/dL, and 350 mg/dL, respectively. In patients with defined cut-off glucose levels of 250 mg/dL and 300 mg/dL, SIH was associated with a 3.5-fold (95% confidence interval (CI) 1.61–7.46; *p* = 0.001) and 3-fold (95% CI 1.11–8.03; *p* = 0.030) higher odds of mortality, adjusted by sex, age, pre-existing comorbidities, and injury severity score, than the 491 patients with diabetic normoglycemia (DN). However, in patients with a defined cut-off glucose level of 350 mg/dL, adjusted mortality in SIH in DM was insignificantly different than that in DM. According to the receiver operating characteristic (ROC) curve analysis, a blood sugar of 233 mg/dL, a glycemic gap of 79 (i.e., blood sugar of 251 mg/dL), and a SHR of 1.45 (i.e., blood sugar of 250 mg/dL) were identified as cut-offs for mortality outcomes, with AUCs of 0.622, 0.653, and 0.658, respectively. *Conclusions:* In this study, a cut-off glucose level of 250 mg/dL was selected to provide a better definition of SIH in DM than glucose levels of 300 mg/dL or 350 mg/dL.

## 1. Introduction

Stress-induced hyperglycemia (SIH) commonly occurs in patients following a major trauma [1,2,3,4]. SIH is attributed to a state of excess hepatic glucose output, diminished insulin production, and insulin resistance in the peripheral tissues, with excessive adrenal cortical output and high circulating levels of cytokines [5,6]. The prevalence of SIH has been reported as 4% [7] and 4.9% [8] of patients with all trauma causes to 7.8% of patients with isolated moderate to severe trauma brain injuries [9] and up to 48.9% of patients with a hip fracture [10]. It is also associated with increased in-hospital mortality, especially in patients without diabetes [7,11,12]. Kerby et al., reported that SIH demonstrated a >2-fold increase in mortality risk; however, diabetic hyperglycemia (DH) was not associated with a significantly high mortality risk (RR 1.47, 95% CI 0.92–2.36) in trauma patients [7]. Our previous study also revealed that SIH had a 3-fold higher odds ratio (OR) of mortality (95% CI 1.96–4.49; *p* < 0.001) than non-diabetic normoglycemia (NDN), in the selected propensity score-matched patient population, controlled by age, sex, pre-existing comorbidities, and injury severity score (ISS) [8]. However, the mortality rate in DH (OR 1.2, 95% CI 0.99–1.38; *p* = 0.065) was insignificantly higher than that in NDN [8].

The reasons why worse mortality outcomes were only observed in patients with SIH, but not with DH, are not clearly understood. In addition, whether these trauma patients with SIH represent a distinct group with differential outcomes, in comparison with those with DH, remains less explored. Furthermore, the findings regarding the association between hyperglycemia and adverse outcomes are inconsistent among acutely ill diabetes patients [13,14]. One explanation is that patients with diabetes are more adapted to a chronic and premorbid hyperglycemic environment than those patients without diabetes, who may not handle acute hyperglycemia in critical illnesses well [15]. Another plausible explanation for the inconsistent results is the lack of consideration of patients’ premorbid hyperglycemia in many reports, especially in patients with diabetes [16,17,18]. Furthermore, SIH and DH are not mutually exclusive entities, as DH patients may have some degree of stress response, invoking their hyperglycemia. However, whether stress might be more responsible for hyperglycemia in patients with diabetes cannot be specifically identified without measuring stress response hormones or catecholamine levels. In addition, the definition of SIH in the literature remains to be explored.

Glycated hemoglobin A1c (HbA1c) is characterized by decreased biological variability, which is relatively unaffected by acute stress response levels [19]. Measurement of the HbA1c level provides an accurate index of chronic glycemic exposure [19,20]. In an international multicenter study, the long-term average glucose levels were estimated by converting HbA1c values into A1c-derived average glucose (ADAG), using the equation, ADAG = ((28.7 × HbA1c) − 46.7) [21,22], which presented a strong correlation between HbA1c and mean plasma glucose levels in the preceding 3 months. Some glycemic variables, after adjusting for ADAG, are useful in the assessment of adverse outcomes in patients with diabetes [16,17,18]. For example, glycemic gap, the admission glucose level minus ADAG, indicates elevated glucose levels in diabetes patients with critical illnesses. Elevated glycemic gaps of >72 mg/dL and 80 mg/dL were associated with adverse outcomes in diabetes patients with pyogenic liver abscess and in the intensive care unit (ICU), respectively [16,23]. In addition, some authors suggested that the stress hyperglycemia ratio (SHR), calculated as the admission glucose level divided by ADAG, could be used as a better biomarker of critical illness than absolute hyperglycemia [24]. Even at relatively low glucose concentrations (<10 mmol/L or approximately 180 mg/dL), SHR identified patients with relative hyperglycemia as highly at risk for critical illness, with or without hyperglycemia [24].

In this study, we used the concept of long-term average glucose levels in trauma patients to define the optimal level of elevated glucose concentration in determining the occurrence of SIH in patients with diabetes. Three definitions of SIH in diabetes mellitus (DM) were applied, based on the assumption that (1) stress causes a similar elevation of glucose, both in patients, with and without diabetes, resulting in the same glycemic gap between the defined SIH level and ADAG in patients, with and without diabetes; (2) stress causes a similar elevation in percentage of glucose, in patients, with and without diabetes, resulting in the same SHR between the defined SIH level and ADAG in patients, with and without diabetes; and (3) the percentage of patients with SIH among patients, with and without diabetes, is the same. The primary hypothesis of this study was that SIH in patients with diabetes would be correlated with worse outcomes than those without. In this study, the primary outcome measurement was mortality, adjusted by sex, age, pre-existing comorbidities, and injury severity score, and the secondary outcomes were hospital length of stay (LOS) and rates of ICU admission.

## 2. Methods

### 2.1. Ethics Statement

The Institutional Review Board of the Kaohsiung Chang Gung Memorial Hospital, a Level I regional trauma center in southern Taiwan [25,26], approved this study before its implementation (reference number 201701332B0) and waived the need for informed consent.

### 2.2. Data Source and Study Population

**Figure 1:**
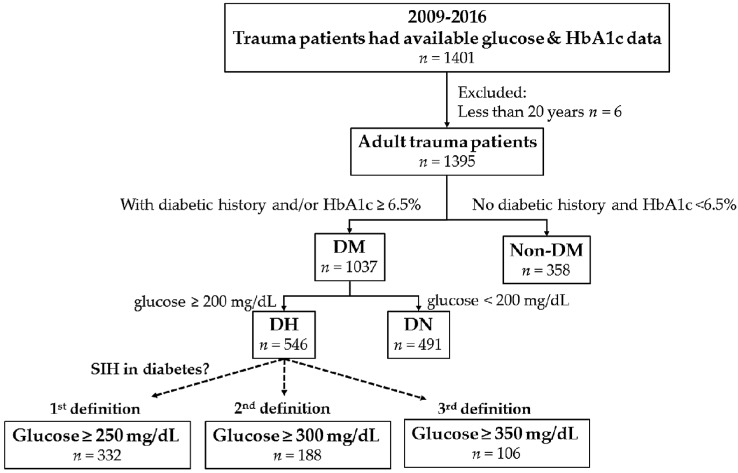
Flow chart to divide patients into diabetes mellitus (DM), non-diabetes mellitus (non-DM), diabetic normoglycemia (DN), diabetic hyperglycemia (DH), and stress-induced hyperglycemia, in diabetes groups, based on various glucose levels, SIH: stress-induced hyperglycemia.

This retrospective study reviewed the data of all hospitalized trauma patients registered in the Trauma Registry System, from 1 January 2009 to 31 December 2016. Only adult patients, aged ≥20 years, with available data on serum glucose and HbA1c levels upon admission, were included in the study (Figure 1). According to the guideline for DM diagnosis in the American Diabetes Association, DM was determined based on patient history and/or a HbA1c level of ≥6.5% [27]. The HbA1c level was based on a measurement within 1 month preceding or following admission. Those patients who had no patient history and admission HbA1c levels of <6.5% were classified as patients without diabetes. Among the patients with diabetes, DH and diabetic normoglycemia (DN) were diagnosed based on serum glucose levels of ≥200 mg/dL and <200 mg/dL upon arrival at the emergency department (ED). Detailed patient information retrieved from the Trauma Registry System included the following: sex; age; comorbidities, such as hypertension (HTN), coronary artery disease (CAD), congestive heart failure (CHF), cerebrovascular accident (CVA), and end-stage renal disease (ESRD); Glasgow Coma Scale (GCS); ISS, expressed as the median and interquartile range (IQR, Q1–Q3); serum glucose level at the ED; HbA1c level; chronic average blood glucose (converted based on the HbA1c level using the equation ADAG = ((28.7 × HbA1c) − 46.7) [20,21]); glycemic gap (calculated using the equation, glycemic gap = (admission glucose − ADAG)); stress hyperglycemia ratio (SHR), calculated as the admission glucose level divided by ADAG; hospital LOS; rates of ICU admission; and in-hospital mortality.

### 2.3. Definition of SIH in DM

Patients with high glucose level were divided into SIH in DM groups, based on the following definitions and comparisons with those of DN: (1) same glycemic gap from ADAG in patients with and without diabetes; (2) same percentage of elevated glucose (i.e., SHR) between the defined SIH level and ADAG, in patients, with and without diabetes; and (3) same percentage as patients with SIH in patients with and without diabetes.

### 2.4. Statistical Analysis

Outcomes, such as in-hospital mortality, hospital LOS, and ICU admission rate were calculated. The unpaired Student’s *t*-test and Mann–Whitney *U*-test were used to analyze normally and non-normally distributed continuous data, respectively, which was reported as mean ± standard deviation. Categorical data were expressed as frequency (%) and compared using two-sided Fisher’s exact or Pearson’s chi-square tests. ORs with 95% CIs of the associated conditions of the patients were presented. Adjusted ORs (AORs) for mortality, adjusted by sex, age, pre-existing comorbidities, and ISS with its 95% CIs, were also calculated. Variables, including blood sugar, glycemic gap and SHR were evaluated for cut-off points that could predict the probability of mortality of diabetes patients by plotting specific receiver operating characteristic (ROC) curves. The accuracy of each parameter in predicting the mortality outcomes was calculated, based on the maximal Youden index for each cut-off point. The maximal Youden index reflects the maximal correct classification accuracy and was calculated as sensitivity + specificity − 1. *p*-Values of <0.05 were defined as statistically significant. All the statistical analyses were performed using IBM SPSS Statistics for Windows version 20.0 (IBM Corp., Armonk, NY, USA).

## 3. Results

### 3.1. Characteristics of Patients with and without Diabetes

Figure 1 shows the enrolled study population, including 1395 adult trauma patients, who were divided into two groups: DM (*n* = 1037), comprising 546 DH and 491 DN patients, and non-DM (*n* = 358). Compared to non-DM, a significant female predominance was observed in the DM group (Table 1). Patients in the DM group were significantly older than those in the non-DM group. Among the pre-existing comorbidities, DM had higher odds of HTN, but not other comorbidities, compared to non-DM. The DM group had a significantly higher GCS score than non-DM. However, the ISS was insignificantly different between DM and non-DM groups, regardless of stratification by an ISS of <16, 16–24, or ≥25. The DM group had significantly higher HbA1c levels (7.6 ± 1.8% vs. 5.7 ± 0.4%, respectively; *p* < 0.001) and calculated ADAG (172.2 ± 51.2 mg/dL vs. 115.4 ± 12.0 mg/dL, respectively; *p* < 0.001) as well as higher glucose levels at the ED (224.3 ± 103.9 mg/dL vs. 144.6 ± 44.5 mg/dL, respectively; *p* < 0.001) than the non-DM group. The DM group also had a significantly higher glycemic gap (52.1 ± 87.9 mg/dL vs. 29.2 ± 43.6 mg/dL, respectively; *p* < 0.001) and SHR (1.3 ± 0.5 vs. 1.3 ± 0.4, respectively; *p* = 0.023) than the non-DM group. Remarkably, based on SHR, the percentage of elevated glucose level at the ED and ADAG was 30% in patients both with and without diabetes. The mortality rate was insignificantly higher in the DM group that that in the non-DM group.

### 3.2. Definition of SIH in DM

**Figure 2:**
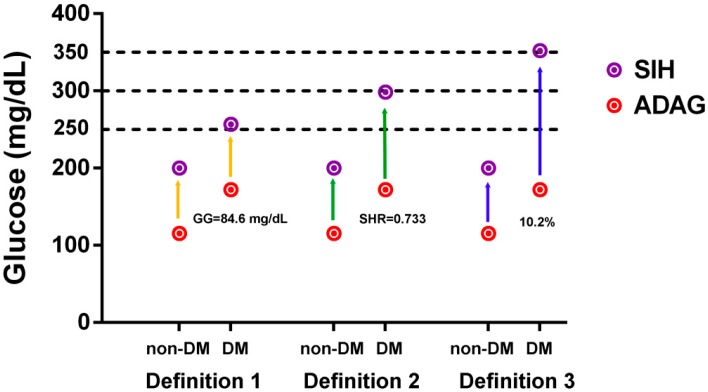
The defined glucose level by three different definitions of SIH in diabetes, based on the assumption that stress causes the same (1) elevation of glucose (glycemic gap); (2) glucose elevation percentage (i.e., SHR); and (3) percentage of patients with SIH, both in patients with and without diabetes.

**Figure 3:**
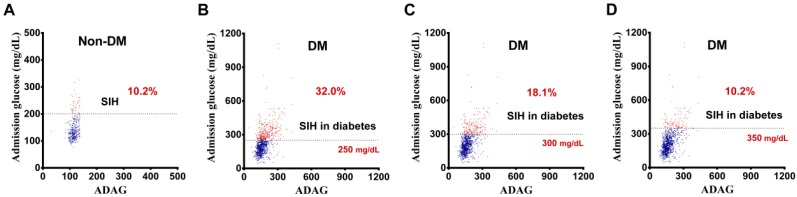
(**A**) The percentage of patients with SIH in non-DM and DM groups, based on defined glucose levels set at (**B**) 250 mg/dL; (**C**) 300 mg/dL; and (**D**) 350 mg/dL.

Figure 2 shows the first definition of SIH in DM, based on the assumption that stress causes a similar elevation of glucose levels, both in patients with and without diabetes, which results in the same glycemic gap between the defined SIH level and ADAG. In patients without diabetes, the glycemic gap between defined SIH (glucose ≥ 200 mg/dL) and ADAG (115.4 mg/dL) was 84.6 mg/dL. Therefore, the defined SIH level in DM was calculated as 172.2 mg/dL (ADAG of DM) + 84.6 mg/dL = 256.8 mg/dL. The second definition of SIH in DM was based on the assumption that stress causes a similar elevation in the percentage of glucose levels, both in patients with and without diabetes, which results in the same glucose elevation percentage (i.e., SHR) between the defined SIH level and ADAG. In patients without diabetes, the SHR between the defined SIH (glucose level of ≥200 mg/dL) and ADAG (115.4 mg/dL) was 0.733, i.e., the elevation of glucose is 73.3% of ADAG. Therefore, the defined SIH level in DM would be 172.2 mg/dL (ADAG of DM) + 126.2 (i.e., 172.2 multiplied to 0.733) mg/dL = 298.4 mg/dL. The third definition of SIH in DM was based on the assumption that the percentage of patients with SIH, in patients with and without diabetes, is the same. Based on this definition, the percentage of patients with SIH in DM would be the same as that in patients without diabetes (10.2%), thus making the defined SIH level in DM, 351.2 mg/dL. Therefore, the glucose values used to define the arbitrary cut-off glucose level in SIH in DM were 250 mg/dL, 300 mg/dL, and 350 mg/dL in the first, second, and third definitions, respectively. Therefore, a total of 332 (32.0%), 188 (18.1%), and 106 (10.2%) patients were classified as diabetes patients with SIH, based on the first, second, and third definitions, respectively (Figure 1 and Figure 3).

### 3.3. Patient Outcomes Based on Different Definitions

**Figure 4:**
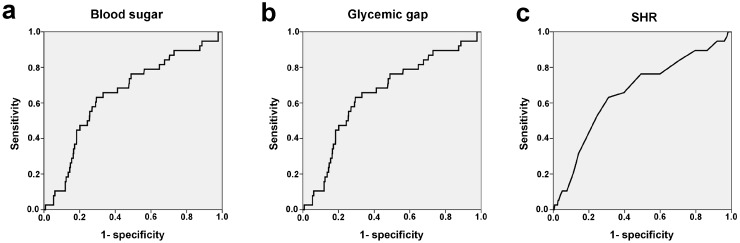
Receiver operating characteristic (ROC) curve analysis to identify cut-off levels for mortality by different parameters, including (**a**) blood sugar (mg/dL), (**b**) glycemic gap (mg/dL) and (**c**) stress hyperglycemia ratio (SHR).

The patients’ profiles in Table 2 and the related statistical analyses in Table 3 show that sex was insignificantly different between DN and SIH in DM, regardless of the definition of glucose level (250 mg/dL, 300 mg/dL, or 350 mg/dL) (Table 2 and Table 3). Compared to DN, patients with SIH in DM in all three definitions were significantly younger and had higher odds of HTN, but lower odds of CVA. However, SIH in DM, based on all three definitions, had a significantly lower GCS score and higher ISS than DN. In the first definition (glucose level of ≥250 mg/dL), the SIH in DM had a 3.1-fold higher odds of mortality (95% CI 1.48–6.48; *p* = 0.002), 3.5-fold higher odds of adjusted mortality (95% CI 1.61–7.46; *p* = 0.001), significantly longer hospital LOS (13.6 days vs. 11.3 days, respectively; *p* = 0.013), and higher proportion of patients admitted to the ICU (33.7% vs. 24.2%, respectively; *p* = 0.003) than DN. In the second definition (glucose level of ≥300 mg/dL), the SIH in DM had a 2.5-fold higher odds of mortality (95% CI 1.02–5.87; *p* = 0.038), 3-fold higher odds of adjusted mortality (95% CI 1.11–8.03; *p* = 0.030), significantly longer hospital LOS (14.2 days vs. 11.3 days, respectively; *p* = 0.013), and higher proportion of patients admitted to the ICU (34.6% vs. 24.2%, respectively; *p* = 0.007) than DN. In the third definition (glucose level of ≥350 mg/dL), the SIH in DM did not have significant differences regarding mortality (OR 1.3; 95% CI 0.35–4.64; *p* = 0.723), adjusted mortality (OR 1.8; 95% CI 0.44–7.14; *p* = 0.427), and hospital LOS (13.4 days vs. 11.3 days, respectively; *p* = 0.091) than DN; however, the proportion of patients admitted to the ICU (34.0% vs. 24.2%, respectively; *p* = 0.038) remained higher than that in DN. According to the ROC curve analysis, a blood sugar of 233 mg/dL, a glycemic gap of 79 (i.e., blood sugar of 251 mg/dL), and a SHR of 1.45 (i.e., blood sugar of 250 mg/dL) were identified as the cut-offs for mortality outcomes, with AUCs of 0.622, 0.653, and 0.658, respectively (Figure 4). However, the discriminating powers of the blood sugar, glycemic gap, and SHR were less satisfied in the conditions where all AUCs were less than 0.70.

## 4. Discussion

This study used the concept of long-term average glucose levels and tried to define the optimal level of elevated glucose concentration in determining the occurrence of SIH in patients with diabetes, according to the clinical outcomes in a broad group of hospitalized hyperglycemic patients. The defined glucose levels were set at 250 mg/dL, 300 mg/dL, and 350 mg/dL, based on three different definitions of SIH in DM under the assumption that stress causes the same (1) elevation of glucose level; (2) glucose elevation percentage; and (3) percentage of patients with SIH, both in patients with and without diabetes. However, the defined glucose level at 350 mg/dL, based on the same percentage of patients with SIH, may not be considered as the cut-off of SIH in DM, because the mortality and adjusted mortality rates were not different between the selected groups of diabetes patients with SIH and those with DN. Mortality rates were consistent with the primary hypothesis that SIH in patients with diabetes would present worse outcomes than those patients without, as these phenomena were observed in non-diabetes patients with SIH, rather than those without. Furthermore, the ROC curve analysis indicated the best cut-off point in predicting mortality of patients with diabetes was a glucose level around 250 mg/dL, which is in accordance with that in the first definition (glucose level of ≥250 mg/dL). However, according to this definition, it remains a concern that 32.0% of the patients with diabetes were associated with SIH, which is far higher than the 10.2% rate of SIH in patients without diabetes. In addition, the second definition that defined the cut-off glucose level in defining SIH in DM was 300 mg/dL. This second definition, based on similar percentages of glucose elevation, both in patients with and without diabetes, is more reasonable, because the admission glucose level is at 1.3-fold of ADAG, in both patients with and without diabetes with a similar severity of injury in this study. Under the hypothesis that stress presumably caused a similar percentage of glucose increase both in patients with and without diabetes, in the second definition (glucose level of ≥300 mg/dL), the SIH in DM had a 3-fold higher odds of adjusted mortality than DN, which is comparable with the 3-fold adjusted mortality of SIH in patients without diabetes, compared to NDN [8].

Although hyperglycemia is reported to be associated with increased morbidity and mortality in trauma patients [1,3,6], the mechanisms for potential detrimental effects of hyperglycemia in SIH and DH may be different, because SIH is an acute process, initiated by the release of stress hormones and cytokines, while DH is a chronic process associated with subsequent microvascular changes, such as CAD, peripheral vascular disease, and nephropathy [6]. However, hyperglycemia in DM, after a traumatic injury or during a critical illness, was not attributed with DM, per se, but may be partially caused by the response to stress. So far, the diagnosis of DH does not preclude a diabetes patient from having a stress-induced hyperglycemic response. This is an important issue that should be addressed in the clinical setting. For example, some authors have proposed the tight glycemic control—the practice of maintaining blood glucose levels between 80 mg/dL and 110 mg/dL using intensive intravenous insulin—based on observational data that has demonstrated an association between hyperglycemia, increased risk of infection, and decreased survival [28]. However, in dealing with SIH in patients without diabetes, we generally do not perform tight glucose control; however, in patients with diabetes, some authors have proposed that tight glycemic control may have a substantial benefit [29]. Interestingly, further evidence has demonstrated that intensive insulin therapy significantly increases the risk of hypoglycemia and confers no overall mortality benefit among critically ill patients [30,31]. The largest multinational randomized clinical trial (the Normoglycemia in Intensive Care Evaluation and Survival Using Glucose Algorithm Regulation (NICE-SUGAR)) examined tight glycemic control in the broadest cohort of surgical and medical ICU patients and demonstrated that tight glycemic control increases the risk of developing severe hypoglycemia and 90-day mortality [30]. Furthermore, a large international randomized trial among adults in the ICU revealed that intensive glucose control of a blood glucose target of 180 mg resulted in lower mortality than did a target of 81–108 mg/dL [28]. These results also indicated the importance of identifying a stress component in inducing hyperglycemia in patients with diabetes. However, because the levels of stress response hormones or catecholamines were measured in this retrospective study, recognizing which parts of hyperglycemia were attributed to the stress response in patients with DH, was not specifically possible. This would be the major limitation in the interpretation of results in this study.

Some other limitations in this study should be acknowledged. First, patients without HbA1c data were excluded because HbA1c is a mandatory component in calculating ADAG; however, HbA1c level was not regularly checked for trauma patients, particularly those who were younger and had no history. Therefore, many non-diabetes patients without HbA1c data would have been excluded from the study, thus making a selection bias through an overestimation of the HbA1c level in the control group. In addition, potential causes of spurious HbA1c results, including abnormal hemoglobin levels and conditions affecting red cell turnover (hemolysis, chronic malaria, major blood loss, and transfusions), were insufficiently justified in this study [19,20]. Second, the retrospective design of the study may have created an inherent selection bias and prevented an estimation of the treatment effects, such as glucose control or drug use. Finally, patients declared dead at the scene of accident or upon hospital arrival were not included in the database, which may have also led to a selection bias.

## 5. Conclusions

According to the ROC curve analysis in predicting mortality, we selected 250 mg/dL as the cut-off glucose level in defining SIH in DM. Based on this definition, the SIH in DM comprised 32.0% of all patients with diabetes and presented a 3.5-fold higher odds of adjusted mortality than those patients with DN after a traumatic injury. However, further effort in providing the measurements of stress response hormones is important, to validate this assumption.

## Figures and Tables

**Table 1:** Characteristics and glycemic variables of patients, with and without diabetes.

Variables	DM	Non-DM
	*n* = 1037	*n* = 358
Sex		
Female, *n* (%)	541 (52.2)	161 (45.0)
Male, *n* (%)	496 (47.8)	197 (55.0)
Age (years)	67.1 ± 12.5	64.4 ± 16.8
Comorbidity		
HTN, *n* (%)	669 (64.5)	168 (46.9)
CAD, *n* (%)	120 (11.6)	33 (9.2)
CHF, *n* (%)	30 (2.9)	11 (3.1)
CVA, *n* (%)	137 (13.2)	43 (12.0)
ESRD, *n* (%)	3 (0.3)	1 (0.3)
GCS	14.1 ± 2.5	13.6 ± 3.2
ISS, median (IQR)	9 (5–14)	9 (5–16)
<16	781 (75.3)	257 (71.8)
16–24	189 (18.2)	67 (18.7)
≥25	67 (6.5)	34 (9.5)
HbA1c (%)	7.6 ± 1.8	5.7 ± 0.4
ADAG	172.2 ± 51.2	115.4 ± 12.0
Blood sugar (mg/dL)	224.3 ± 103.9	144.6 ± 44.5
Glycemic gap (mg/dL)	52.1 ± 87.9	29.2 ± 43.6
SHR	1.3 ± 0.5	1.3 ± 0.4
Mortality, *n* (%)	38 (3.7)	16 (4.5)

ADAG = HbA1c-derived average glucose; CAD = coronary artery disease; CHF = congestive heart Failure; CI = confidence interval; CVA = cerebral vascular accident; DM = diabetes mellitus; ESRD = end-stage renal disease; HbA1c = hemoglobin A1c; HTN = hypertension; ICU = intensive care unit; IQR = interquartile range; ISS = injury severity score; LOS = length of stay; OR = odds ratio; SHR = stress hyperglycemia ratio.

**Table 2:** Characteristics, injury severities, and outcomes of SIH in diabetes based on defined glucose levels set at 250 mg/dL, 300 mg/dL, and 350 mg/dL as well as patients with diabetic normoglycemia.

Variables	(I)	(II)	(III)	(IV)
	Glucose ≥ 250	Glucose ≥ 300	Glucose ≥ 350	DN
	*n* = 332	*n* = 188	*n* = 106	*n* = 491
Sex				
Female, *n* (%)	166 (50.0)	91 (48.4)	55 (51.9)	250 (50.9)
Male, *n* (%)	166 (50.0)	97 (51.6)	51 (48.1)	241 (49.1)
Age (years)	64.6 ± 13.7	63.6 ± 13.7	63.2 ± 14.1	68.5 ± 11.5
Comorbidity				
HTN, *n* (%)	187 (56.3)	106 (56.4)	62 (58.5)	340 (69.2)
CAD, *n* (%)	33 (9.9)	16 (8.5)	7 (6.6)	64 (13.0)
CHF, *n* (%)	10 (3.0)	7 (3.7)	5 (4.7)	12 (2.4)
CVA, *n* (%)	27 (8.1)	15 (8.0)	9 (8.5)	79 (16.1)
ESRD, *n* (%)	2 (0.6)	2 (1.1)	1 (0.9)	1 (0.2)
GCS	13.6 ± 3.3	13.6 ± 3.2	13.6 ± 3.2	14.4 ± 2.0
ISS, median (IQR)	9 (9–16)	9 (6–16)	9 (9–16)	9 (5–10)
<16	223 (67.2)	124 (66.0)	70 (66.0)	389 (79.2)
16–24	74 (22.3)	46 (24.5)	27 (25.5)	81 (16.5)
≥25	35 (10.5)	18 (9.6)	9 (8.5)	21 (4.3)
Mortality, *n* (%)	22 (6.6)	10 (5.3)	3 (2.8)	11 (2.2)
Adjusted mortality, *n* (%)	22 (6.6)	10 (5.3)	3 (2.8)	11 (2.2)
Hospital LOS (days)	13.6 ± 13.5	14.2 ± 13.9	13.4 ± 11.1	11.3 ± 11.2
ICU admission, *n* (%)	112 (33.7)	65 (34.6)	36 (34.0)	119 (24.2)

CAD = coronary artery disease; CHF = congestive heart Failure; CI = confidence interval; CVA = cerebral vascular accident; DM = diabetes mellitus; DN = diabetic normoglycemia; ESRD = end-stage renal disease; HTN = hypertension; ICU = intensive care unit; IQR = interquartile range; ISS = injury severity score; LOS = length of stay.

**Table 3:** Comparison between SIH in diabetes based on defined glucose levels against the patients with diabetic normoglycemia.

Variables	(I) vs. (IV)	(II) vs. (IV)	(III) vs. (IV)
	OR (95% CI)	OR (95% CI)	OR (95% CI)
Sex	0.796	0.558	0.856
Female, *n* (%)	1.0 (0.73–1.27)	0.9 (0.65–1.27)	1.0 (0.68–1.58)
Male, *n* (%)	1.0 (0.79–1.37)	1.1 (0.79–1.55)	1.0 (0.63–1.46)
Comorbidity			
HTN, *n* (%)	0.6 (0.43–0.77)	0.6 (0.41–0.81)	0.6 (0.41–0.96)
CAD, *n* (%)	0.7 (0.47–1.15)	0.6 (0.35–1.10)	0.5 (0.21–1.06)
CHF, *n* (%)	1.2 (0.53–2.90)	1.5 (0.60–3.98)	2.0 (0.68–5.73)
CVA, *n* (%)	0.5 (0.29–0.73)	0.5 (0.25–0.81)	0.5 (0.24–1.00)
ESRD, *n* (%)	3.0 (0.27–32.88)	5.3 (0.48–58.45)	4.7 (0.29–75.21)
ISS			
<16	0.5 (0.39–0.74)	0.5 (0.35–0.74)	0.5 (0.32–0.81)
16–24	1.5 (1.02–2.06)	1.6 (1.09–2.47)	1.7 (1.05–2.85)
≥25	2.6 (1.51–4.62)	2.4 (1.23–4.56)	2.1 (0.92–4.67)
Mortality, *n* (%)	3.1 (1.48–6.48)	2.5 (1.02–5.87)	1.3 (0.35–4.64)
Adjusted mortality, *n* (%)	3.5 (1.61–7.46)	3.0 (1.11–8.03)	1.8 (0.44–7.14)
ICU admission, *n* (%)	1.6 (1.17–2.16)	1.7 (1.15–2.38)	1.6 (1.02–2.53)

CAD = coronary artery disease; CHF = congestive heart Failure; CI = confidence interval; CVA = cerebral vascular accident; DM = diabetes mellitus; ESRD = end-stage renal disease; HTN = hypertension; ICU = intensive care unit; IQR = interquartile range; ISS = injury severity score; LOS = length of stay; OR = odds ratio.
